# New insights into the prognostic value of Ki-67 labeling index in patients with triple-negative breast cancer

**DOI:** 10.18632/oncotarget.8531

**Published:** 2016-04-01

**Authors:** Shuang Hao, Zhi-Xian He, Ke-Da Yu, Wen-Tao Yang, Zhi-Min Shao

**Affiliations:** ^1^ Department of Breast Surgery, Fudan University Shanghai Cancer Center, Shanghai, China; ^2^ Department of Oncology, Shanghai Medical College, Fudan University, Shanghai, China; ^3^ Department of General Surgery, Affiliated Hospital of Nantong University, Nantong, Jiangsu, China; ^4^ Department of Pathology, Fudan University Shanghai Cancer Center, Shanghai, China; ^5^ Institutes of Biomedical Science, Fudan University, Shanghai, China; ^6^ Key Laboratory of Breast Cancer in Shanghai, Department of Breast Surgery, Fudan University Shanghai Cancer Center, Shanghai, China

**Keywords:** triple-negative breast cancer, Ki67 labeling index, breast-cancer specific survival, heterogeneous

## Abstract

The clinicopathological importance of the Ki-67 labeling index (LI) in breast cancer has been studied intensely; however, its prognostic significance in triple-negative breast cancer (TNBC) is unclear. We aimed to determine the optimal Ki-67 cut-off point to demonstrate its prognostic relevance for breast-cancer-specific survival (BCSS) in TNBC patients. A total of 571 female TNBC patients underwent diagnosis and surgery at our institution from January 2002 to June 2011. Clinicopathological information for all patients was available and categorized by Ki-67 LI and age at diagnosis. The cut-off values for Ki-67 LI and age were selected using the medians. A varying-coefficient Cox model was used to describe the effect of Ki-67 LI on BCSS outcomes changing with age after adjustment for disease characteristics. For survival analysis, the Kaplan–Meier method and the log-rank test were used. Cox proportional hazards models were applied to determine the association of Ki-67 LI and age with BCSS outcomes after adjustment for disease characteristics. Median age was 50 years, and median Ki-67 LI was 35% (range, 0 – 97.5%). There was no prognostic significance of stratification by Ki-67 LI in all patients. When analyzing age at diagnosis as a continuous variable, the log-transformed HR_Ki67 > 35% vs. ≤ 35%_ for BCSS increased in an S-shaped curve with increasing age up to about 50 years-old and remained higher-risk for high Ki-67 LI. After adjusting for clinicopathological risk factors, low Ki-67 LI was a poor prognostic factor for BCSS (HR: 0.36, 95% CI: 0.14–0.96, *P* = 0.042) in patients of ≤ 50 years, but not in patients diagnosed at > 50 years (hazard ratio [HR]: 1.57, 95% CI: 0.76–3.22, *P* = 0.241). In conclusion, lower Ki-67 LI has poor prognosis relevance in TNBC patients diagnosed at ≤ 50 years-old. Further validation of the clinical significance of Ki-67 LI in TNBC is required.

## INTRODUCTION

Triple-negative breast cancer (TNBC), as defined by the absence of estrogen receptor (ER), progesterone receptor (PR) and human epidermal growth factor receptor 2 (HER2) expression, corresponds to 10%–20% of all breast cancers [1]. This heterogeneous disease is generally more aggressive, with higher risk of relapse and poorer prognosis as a result of metastatic disease [2, 3]. The limits of known biomarkers have restricted the development of targeted therapy approaches in TNBC [4].

As a proliferation marker, Ki-67 expression varies throughout G1, S, G2, and M cell cycle phases, but not during the G0 resting phase [5]. It has been explored in breast cancer and other malignant diseases. In TNBC, higher Ki-67 expression was strongly predictive of poor overall survival, even with a higher pathological complete response (pCR) rate to neoadjuvant chemotherapy [3, 6, 7]. Despite the large number of studies of Ki-67labeling index (LI), there is still no consensus for the cut-off points of Ki-67 LI, especially for TNBC. TNBC is a heterogeneous disease with distinct molecular subtypes that display unique biology and outcomes, which may lead to variation in the significance of Ki-67 LI.

In this study, we assessed the role of Ki-67 LI in TNBC, and determined the optimal Ki-67 cut-off point to demonstrate its prognostic relevance for breast cancer-specific survival (BCSS).

## RESULTS

### Patient characteristics

The baseline characteristics of the study patients are presented in Table 1. The median age was 50 years (range, 23–86 years), and the overall 5-year BCSS rate was 83%. Since median Ki-67 LI was 35% (range, 0–97.5%), patients were divided in low (≤ 35%) and high (> 35%) Ki-67 LI groups containing 329 (57.6%) and 242 (52.4%) patients, respectively. The high Ki-67 LI group had a significantly higher proportion grade III tumors (*P* < 0.001; Table 1).

**Table 1:** Baseline characteristic of 571 patients

Characteristicsa	Total	Ki-67 ≤ 35%	Ki-67 > 35%	*P*-value
	*N* (%)	*N* (%)	*N* (%)	
Diagnosed age				
≤ 50	286 (50.1%)	151 (45.9%)	(5.8%)	0.120
> 50	285 (49.9%)	178 (54.1%)	107 (44.2%)	
Tumor size				
T1	237 (42.2%)	131 (40.4%)	106 (44.5%)	0.187
T2–3	325 (57.8%)	193 (59.6%)	132 (55.5%)	
Tumor grade				
I–II	242 (44.1%)	165 (52.5%)	77 (32.8%)	< 0.001
III	307 (55.9%)	149 (47.5%)	158 (67.2%)	
Nodal status				
(−)	339 (59.4%)	186 (56.5%)	153 (63.2%)	0.064
(+)	232 (40.6%)	143 (43.5%)	89 (36.8%)	
Chemothearpy				
No	33 (5.8%)	23 (7.0%)	10 (4.1%)	0.102
Yes	538 (94.2%)	306 (93.0%)	232 (95.9%)	

a The numbers of tumor size, tumor grade and nodal status were less than the total number of subjects because some clinical data were missing. The missing data of tumor size and tumor grade is 9 and 22, respectively.

### Ki-67 LI and BCSS

**Figure 1:**
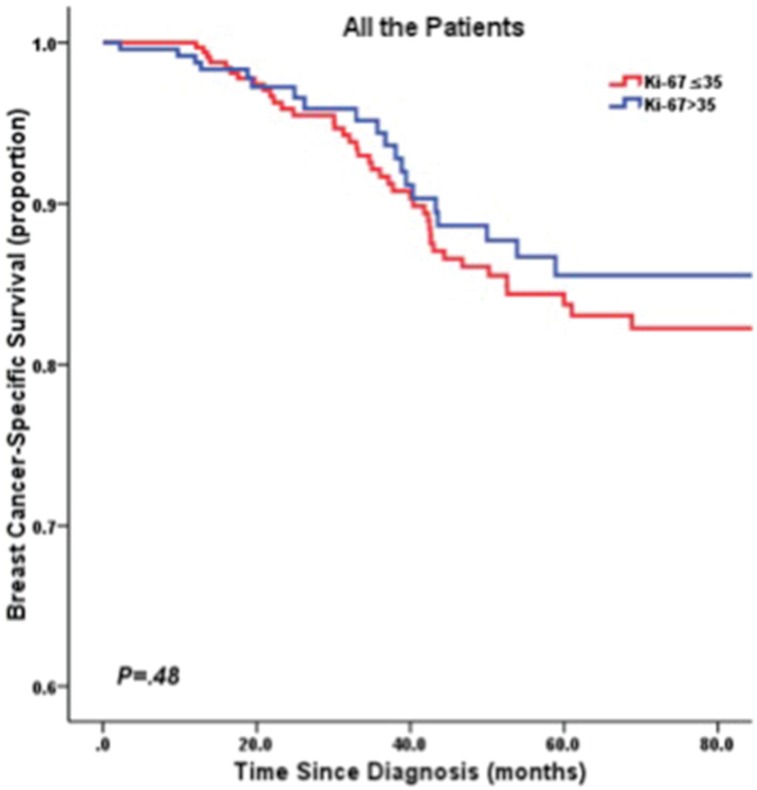
Kaplan-Meier curve of BCSS by Ki67 LI in all TNBC patients

In the overall analysis, we did not find any significant association between Ki-67 LI and BCSS (*P* = 0.481 and *P* = 0.513, respectively; Table 2). Figure 1 presents the corresponding Kaplan–Meier survival curves of BCSS categorized by Ki-67 LI of patients. All clinical and histopathological parameters (age at diagnosis, tumor size, nodal status, tumor grade, and chemotherapy) were also investigated for their prognostic value in a univariate analysis of BCSS. Tumor size (*P* = 0.035) and nodal status (*P* < 0.001) had statistically significant prognostic effects on BCSS (Table 2). In multivariate analysis, only nodal status was significantly correlated with BCSS after adjustment for clinicopathological risk factors (hazard ratio [HR]:5.53, 95% confidence interval [CI]: 2.97–10.29, *P* < 0.001; Table 2).

**Table 2:** Univariate and multivariate BCSS analysis in TNBC patients

Characteristic	Univariate	95% CI	*P*	Multivariate		*P*^a^
	HR			HR^a^	95% CI	
Age						
≤ 50	1.00			1.00		
> 50	1.21	0.73–2.00	0.468	1.16	0.69–1.95	0.587
Ki67 LI (%)						
≤ 35	1.00			1.00		
> 35	0.82	0.48–1.41	0.481	0.83	0.48–1.44	0.513
Tumor size						
T1	1.00			1.00		
T2–3	1.81	1.04–3.15	0.035	1.43	0.82–2.52	0.210
Tumor grade						
I–II	1.00			1.00		
III	0.97	0.58–1.64	0.918	0.95	0.56–1.61	0.852
Nodal status						
(−)	1.00			1.00		
(+)	5.68	3.07–10.51	< 0.001	5.53	2.97–10.29	< 0.001
Chemothearpy						
No	1.00			1.00		
Yes	0.56	0.24–1.30	0.176	0.56	0.24–1.32	0.188

BCSS: breast cancer-specific survival; BMI: body mass index; CI: confidence interval; HR: hazard ratio.

a Adjustedfor Ki67 LI, age at diagnosis, tumor size, nodal status, grade and systemic adjuvant therapy.

### Stratification analysis by age

**Figure 2:**
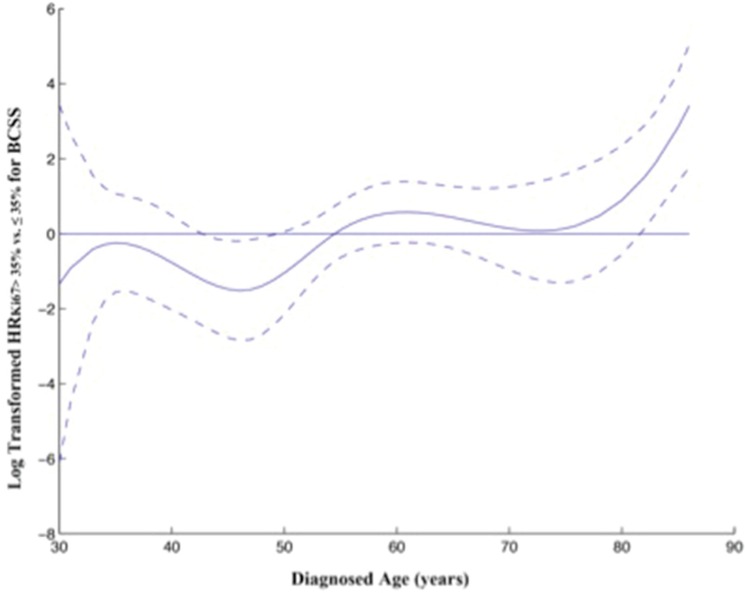
Relationship between diagnosed age and ratio of HR for BCSS stratified by Ki67 LI Thesolidcurve represents the continuous relationship between diagnosed age and the log-transformed HR _Ki67 > 35% vs. ≤ 35%_ for BCSS, based on a varying-coefficient Cox model. Dotted curves represent 95% confidence intervals. HR was adjusted for diagnosed age, tumor size, nodal status and tumor grade.

When analyzing age of diagnosis as a continuous variable, the log-transformed HR_Ki67 > 35% vs. ≤ 35%_ for BCSS increased in an S-shaped curve with increasing age up to about 50 years-old and remained higher-risk for high Ki-67 LI (Figure 2).

**Figure 3:**
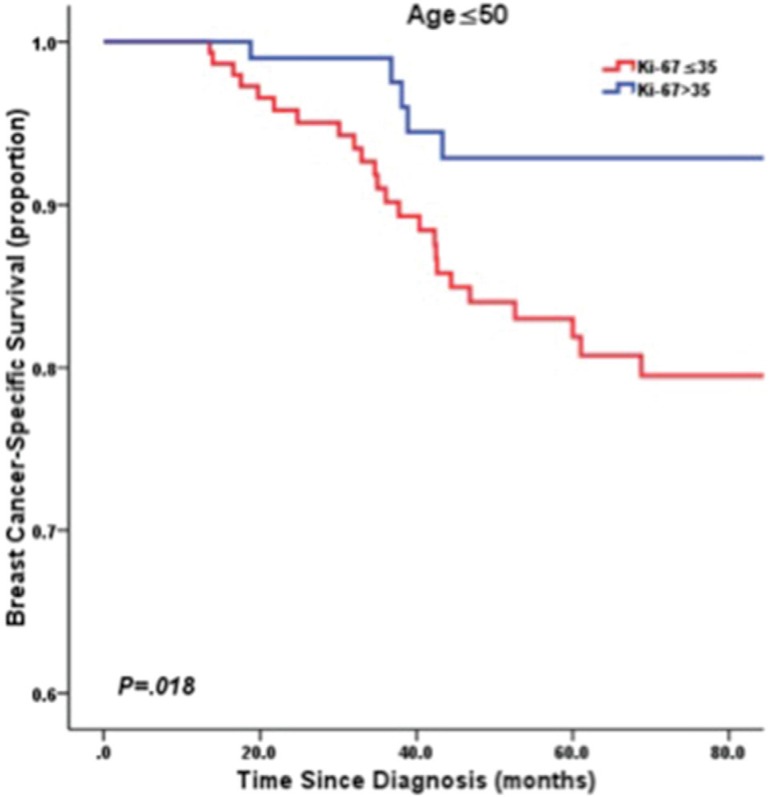
Kaplan-Meier curve of BCSS by Ki67 LI in patients ≤ 50 years old

**Figure 4:**
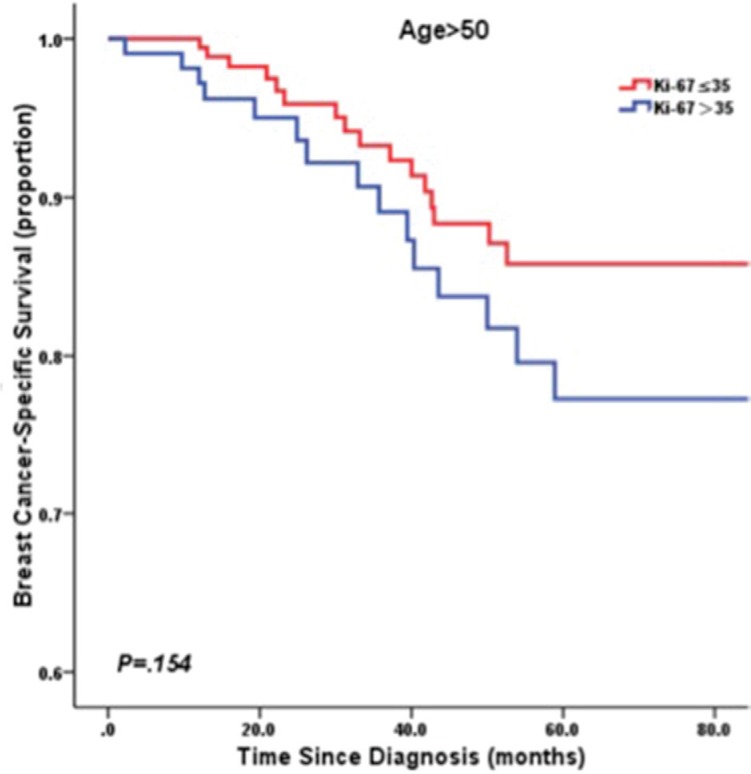
Kaplan-Meier curve of BCSS by Ki67 LI in patients > 50 years old

Accordingly, after stratification by age at diagnosis of 50 years, univariate survival analysis using the Kaplan–Meier method indicated low Ki-67 LI was significantly associated with poorer BCSS in young patients (≤ 50 years) (*P* = 0.018; Figure 3). However, in the patients diagnosed at > 50 years-old, low Ki-67 LI revealed a better prognosis without significance (*P* = 0.154; Figure 4). In multivariate analysis, low Ki-67 LI was a poor prognostic factor for BCSS (HR: 0.36, 95% CI: 0.14–0.96, *P* = 0.042) in patients ≤ 50 years-old after adjustment for clinicopathological risk factors (Table 3). Among the TNBC cases diagnosed at age > 50 years-old, Ki-67 LI did not predict for BCSS (HR: 1.57, 95% CI: 0.76–3.22, *P* = 0.241;Table 3).

**Table 3:** Multivariate survival analysis in TNBC patients according to diagnosed age

Diagnosed age	Total	BCSS	95% CI	*P*^a^
	N (%)	HR^a^		
**Diagnosed age ≤ 50**	286 (50.1%)			
Ki67 LI (%)				
≤ 35	151	1.00		
> 35	135	0.36	0.14–0.96	0.042
Tumor size				
T1	117	1.00		
T2–3	161	1.77	0.78–4.01	0.175
Tumor grade				
I–II	117	1.00		
III	156	1.03	0.49–2.19	0.936
Nodal status				
(−)	170	1.00		
(+)	116	7.08	2.68–18.6	< 0.001
Chemothearpy				
No	7	1.00		
Yes	279	0.58	0.20–1.43	0.236
**Diagnosed age > 50**	285 (49.9%)			
Ki67 LI (%)				
≤ 35	178	1.00		
> 35	107	1.57	0.76–3.22	0.241
Tumor size				
T1	120	1.00		
T2–3	164	1.12	0.52–2.44	0.769
Tumor grade				
I–II	125	1.00		
III	151	0.99	0.48–2.04	0.986
Nodal status				
(−)	169	1.00		
(+)	116	4.49	1.96–10.29	< 0.001
Chemothearpy				
No	26	1.00		
Yes	259	0.37	0.15–0.92	0.031

a Adjustedfor Ki67 LI, age at diagnosis, tumor size, nodal status, grade and systemic adjuvant therapy.

## DISCUSSION

In TNBC, it was found that Ki-67 levels were significantly increased compared with other histological types. Its expression also represented a direct correlation with tumor size and grade in TNBC patients, and higher levels (> 35% staining) were linked with an increased risk of death. Ki-67 accumulation was also associated with a higher pCR to chemotherapy but poor RFS and OS in TNBC [8]. However, data on the relationship between Ki-67 LI and TNBC are limited, especially in different age groups.

In the current study, 571 cases were recruited and divided into two groups according to age above and below 50 years old. Interestingly, our result showed that in young patients with TNBC, low Ki-67 indicated poor prognosis, while in the elderly it denoted good prognosis but without statistical significance.

In the current literature, there are limited and conflicting data with regard to the prognostic impact of age in patients with TNBC. In a previous study of patients with metastatic TNBC, age at diagnosis of less than 50 years was an independent adverse prognostic factor in multivariate analysis [9]. One potential reason could be that the awareness of both patients and physicians of breast cancer may be greater around menopause than in very young women [10]. This effect and higher mammographic breast density at a young age could lead to delayed diagnosis and an advanced stage of the disease. However, the observed effect of young age on prognosis persisted upon multivariate analysis taking such variables into account. Moreover, differences in breast density seemed to not affect the final outcome [11]. Thus, biological differences may underlie the distinct prognosis in very young patients compared with older women.

Based on gene ontologies and differential gene expression, TNBC is classified into seven subtypes: basal-like 1 (BL1), basal-like 2 (BL2), immunomodulatory (IM), mesenchymal (M), mesenchymal stem-like (MSL), luminal androgen receptor (LAR), and unstable (UNS) [12]. Among these subtypes, BL is more often observed in young patients with aggressive tumor behavior, while LAR is generally identified in the elderly [13] with adverse clinical outcome [14]. Each of them harbors a dominant biological function/pathway, which could justify the above distinction. For example, BL subtypes are characterized by high proliferation rates with average Ki-67 staining of 70% in addition to high expression of genes involved in the cell cycle and cell division [15]. It is to be noted that a Ki-67 LI of > 35% is selected as a surrogate marker for BL1 and BL2 chemotherapy, [16] as it can predict chemotherapy-response and improve survival. The M subtype displays higher expression of proliferation-associated genes, including Ki-67, while the MSL subtype expresses these genes at low levels [12]. Testing for AR and PI3K activity is suggested to be reserved for patients with a Ki-67 LI of < 35%, given the low proliferation rate in LAR and mesenchymal-like subtypes [17].

TNBC should be viewed as a group of different diseases that have similar phenotypes but different genotypes with distinct molecular characteristics. In view of this, TNBC can be considered an excellent example of the value of tailored therapy [17]. A more logical approach should consider upfront stratification of TNBC patients based on genetic or surrogate IHC biomarkers that reflect various subtypes and driving pathways to allow treatment to be personalized according to the intrinsic tumor signature. Ideally, stratifying patients based on genetic testing represents the optimal approach.

This study has several limitations. Because of the retrospective nature of the study, we were not able to present data regarding the classification of TNBC based on gene expression signatures. Another possible limitation could be the heterogeneity of the adjuvant treatment. In addition, the current study is based on single institution data, and could be biased in terms of patient selection. Future well-designed and genomic studies are warranted to confirm these findings, and to uncover the mechanism of the molecular characteristics of patients with TNBC.

In conclusion, this study indicates the prognostic role of Ki-67 LI in different age groups of patients with TNBC. In the young, high Ki-67 LI is a good independent prognostic factor for BCSS, and these patients may benefit from chemotherapy with agents that engage DNA damage signaling response pathways. While in older populations, low Ki-67 LI may predict a positive result. In the future, more studies are needed to determine whether prognostic factors, useful biomarkers and/or more complex molecular signatures will contribute to the development of innovative classifications that reflect the clinical behavior of each TNBC subtype and consequently guide treatment choices.

## METHODS

### Study population

A total of 968 women who received treatment for early-stage TNBC from January 2002 to June 2011 were identified. All cases were histopathologically confirmed as TNBC with no distant metastasis at initial diagnosis. Patients were excluded if they received neoadjuvant chemotherapy (*n* = 118), without sufficient follow-up data (*n* = 101) or Ki-67 LI information (*n* = 178), resulting in 571 patients being included in this study. These patients were inhabitants of the administrative regions of eastern China (including Shanghai city, Zhejiang province, Jiangsu province and the surrounding areas). All patients at risk of relapse received adjuvant chemotherapy, using different regimens according to the standards used at the time of surgery, followed by radiotherapy if required. A total of 146 (25.6%) patients received radiotherapy. Clinical information was extracted from the archival medical records. Information on date and cause of death was obtained from local Center of Disease Control records in October 2013. The study was conducted according to the principles expressed in the Declaration of Helsinki and approved by the institutional review board of Fudan University Shanghai Cancer Center. All patients enrolled in this study signed the informed consent voluntarily.

### Immunohistochemistry

In the current study, all cases were histopathologically confirmed independently by two experienced pathologists according to ASCO/CAP 2010 criteria. We reviewed the following histopathological parameters: estrogen receptor (ER) status; progesterone receptor (PR) status; and expression of c-ERBB-2 and Ki-67 of the selected specimens specifically for the current study. Baseline histopathological parameters were evaluated by immunohistochemical (IHC) analysis using surgical specimens as part of routine clinical practice. ER and PR antibodies were purchased from Dako (clones ER 1D5 1:35 and PR 636 1:50) and were evaluated by an avidin–biotin peroxidase complex assay as described by Shimada et al. [18]. ER and PR were considered positive in breast cancer cells if the positive nuclei number was ≥ 1% [19]. Cytoplasmic staining was ignored [20]. Overexpression of HER2 protein was evaluated using a monoclonal antibody (1:400, Clone PN2A; Dako) and a peroxidase–antiperoxidase technique. Positive HER2 was defined as complete membrane staining in > 10% of tumor cells, [21] using a qualitative HercepTest scale of 0–3+, in which scores 0–1 were negative, and 3 was positive [22]. Fluorescence *in situ* hybridization tests were used when the IHC results were ambiguous (i.e., 2+), or for patients who could not be defined as HER2-positive. Ki-67 expression was quantified using a visual grading system and was determined by counting 1,000 tumor cells using the Olympus Image Analyzer (magnification 400 ×) at the hot spots or at the periphery of the invasive component, and was expressed as the percentage of positive tumor cells.

### Statistical analysis

BCSS was determined as the interval between the initiation of primary surgery and the date of breast cancer-related death. For patients without breast cancer-related death at the time of analysis, the date of the last follow-up (December 2012) was considered right censored. Associations between Ki-67 LI and other characteristics were analyzed using the chi-square test. A varying-coefficient Cox model was used to describe the effect of Ki-67 on BCSS outcomes changing with age at diagnosis after adjustment for disease characteristics [23]. Cubic splines were used to fit the nonparametric function f(Age) of the age of diagnosis. The varying-coefficient Cox model is described as follows:

h (*age*, X) = h_0_ (age) exp (f (Age) × ki67group + n + t + grade)

For the assessment of the influence of Ki-67 LI on survival outcome, we adjusted for tumor size, lymph node status, grade and systemic adjuvant therapy by multivariate Cox proportional hazards models. All *P* values were two-sided. Statistical analyses were carried out using SAS software (SAS Institute, Cary, NC) and R software (http://cran.r-project.org/). *P* values of < 0.05 were considered statistically significant.
